# Brown tumor causing pathologic fracture of the ipsilateral femur and pelvis in a pregnant woman: a case report

**DOI:** 10.1093/jscr/rjaf160

**Published:** 2025-03-26

**Authors:** Kyle Gouveia, Marc D Bouchard, Philip Hache, James R Yan, Snezana Vukmirovic-Popovic, Salem Alowami, Herman Johal

**Affiliations:** Division of Orthopaedic Surgery, Department of Surgery, McMaster University, 1280 Main St W, Hamilton, ON, L8S 4L8, Canada; Faculty of Medicine and Health Sciences, Royal College of Surgeons in Ireland, 123 St Stephen's Green, Dublin 2, D02 YN77, Ireland; Department of Orthopaedics, Faculty of Medicine, University of British Columbia, 2775 Laurel Street, Vancouver, BC, V5Z 1M9, Canada; Division of Orthopaedic Trauma, Vancouver General Hospital, 899 W 12th Ave, Vancouver, BC, Canada; Division of Orthopaedic Surgery, Department of Surgery, McMaster University, 1280 Main St W, Hamilton, ON, L8S 4L8, Canada; St. Joseph’s Healthcare Hamilton, Department of Surgery, 50 Charlton Ave E, Hamilton, ON L8N 4A6, Canada; Division of Clinical Pathology, Department of Pathology and Molecular Medicine, McMaster University, 1280 Main St W, Hamilton, ON, L8S 4L8, Canada; Division of Clinical Pathology, Department of Pathology and Molecular Medicine, McMaster University, 1280 Main St W, Hamilton, ON, L8S 4L8, Canada; Division of Orthopaedic Surgery, Department of Surgery, University of Calgary, 3330 Hospital Dr NW, Calgary, AB, T2N 2T8, Canada; Division of Orthopaedic Trauma, Foothills Medical Centre, 1403 - 29 Street NW, Calgary, AB, T2N 2T9, Canada

**Keywords:** brown tumor, primary hyperparathyroidism, pregnancy, pathologic fracture, bone lesion, multidisciplinary care

## Abstract

We present the case of a 30-year-old pregnant woman who was found to have an aggressive-appearing osteolytic lesion of the left distal femur in the setting of hypercalcemia. Biopsy confirmed a brown tumor secondary to hyperparathyroidism. She underwent a successful parathyroidectomy followed by a Cesarean section. Postpartum, she sustained pathologic fractures of the ipsilateral femur and pelvis due to a fall, requiring operative fixation. She progressed to uncomplicated healing following surgical management. Although brown tumors can appear aggressive on imaging, they typically resolve following treatment of the underlying hyperparathyroidism. Pathologic fractures should be managed according to standard orthopedic principles. Clinicians should include brown tumors in the differential diagnosis when evaluating osteolytic lesions, particularly in the presence of hypercalcemia.

## Introduction

Brown tumors, or osteitis fibrosa cystica, are not true neoplasms but a manifestation of abnormal bone metabolism due to excessive osteoclastic activity in prolonged hyperparathyroidism [1]. These lesions are commonly linked to primary or secondary hyperparathyroidism and may regress following treatment of the underlying condition [2]. Persistent parathyroid hormone (PTH) elevation causes osteoclast-mediated bone resorption, leading to microfractures and hemorrhagic cystic spaces that progressively coalesce. The reddish-brown appearance of these lesions is due to hemosiderin deposition in the osteolytic cavities [2].

Brown tumors may occur in multiple bones, often misdiagnosed as skeletal metastases [3, 4]. They most commonly affect the craniofacial bones, long bones, pelvis, metacarpals, and ribs [5]. Similar to metastatic bone disease, they can weaken bone structure and increase the risk of pathological fracture [6]. Comprehensive diagnostics, including imaging, serum calcium, PTH, alkaline phosphatase, and bone scans, are crucial to assess for parathyroid adenomas. Pregnant patients require a tailored diagnostic approach to minimize fetal risk while managing hyperparathyroidism [7].

Given their potential for misdiagnosis as malignant bone lesions and the risk of pathologic fractures, recognizing brown tumors is essential for orthopedic surgeons. Timely diagnosis and intervention are key for effective management.

## Case report

**Figure 1:**
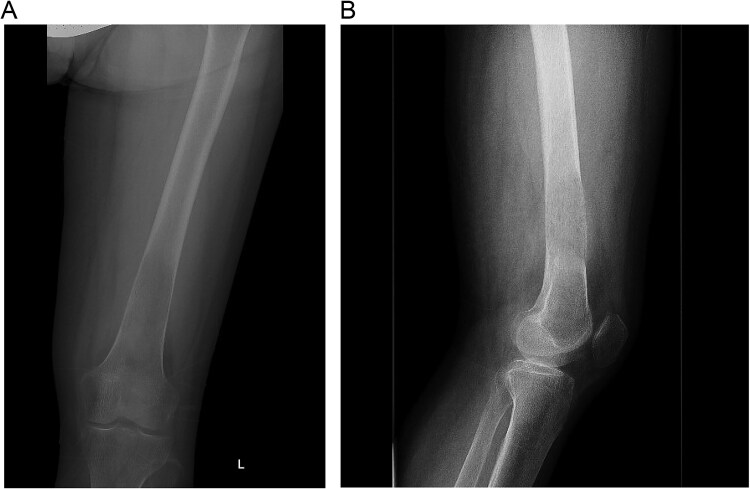
(A and B) Initial radiographs of the distal femur lesion.

A 30-year-old pregnant woman (G2A1) presented at 32 weeks’ gestation with hypertension and left thigh pain, unable to weight-bear for a week. Initial workup included an ultrasound to rule out deep vein thrombosis and radiographs, which showed an aggressive lesion in the distal femur with periosteal reaction (Fig. 1A and B). She was referred to orthopedic oncology, and a biopsy was arranged. Routine blood tests revealed markedly elevated serum calcium of 4.13 mmol/L (normal: 2.10–2.60 mmol/L). Histopathological analysis showed multinucleated giant cells, but radiographs were inconsistent with giant cell tumor (GCT), raising concern for a malignant lesion. Further investigation confirmed primary hyperparathyroidism, prompting consideration of a brown tumor. The patient was referred to head and neck surgery for parathyroidectomy evaluation.

**Figure 2:**
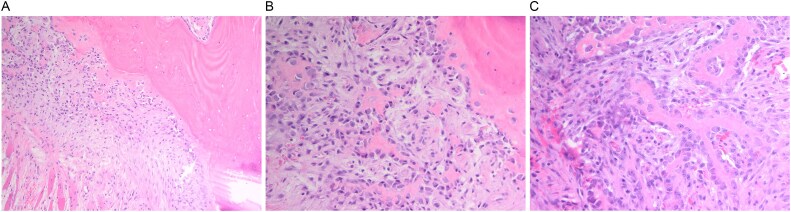
(A–C). Histopathologic images from biopsy specimen at 100× and 200× magnification, showing numerous multinucleated giant cells and mononuclear stromal cells with marked resorption of bone trabeculae and clustering of osteoclasts.

**Figures 3:**
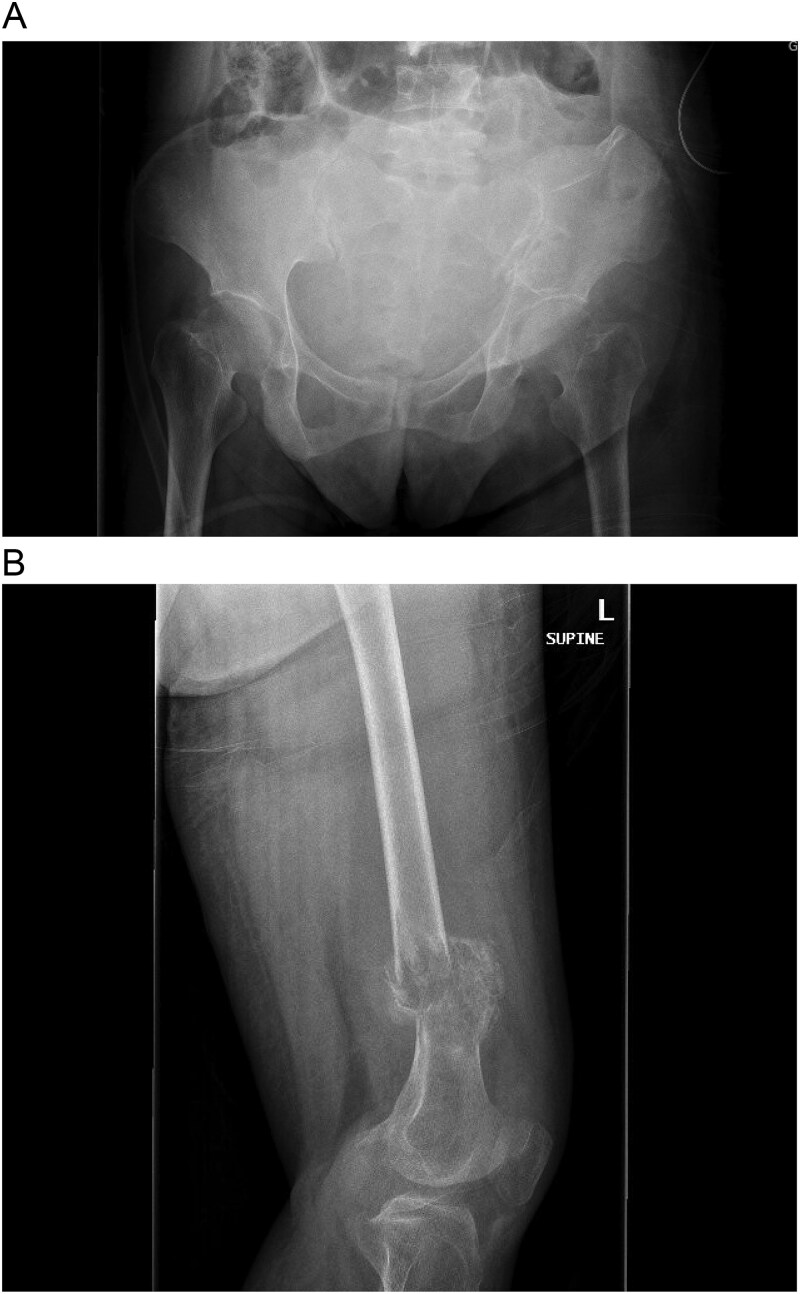
(A and B) Pathologic fracture involving the left distal femur and left iliac bone.

A multidisciplinary discussion involving orthopedic, maternal-fetal medicine, and head and neck teams determined the treatment plan. Further biopsy confirmed the brown tumor diagnosis, showing multinucleated giant cells, mononuclear stromal cells, bone resorption, and osteoclast clustering (Fig. 2A–C). Six days after the presentation, she was deemed suitable for parathyroidectomy. Although at high risk for pathologic fracture, she declined prophylactic fixation during pregnancy. Two days after a successful parathyroidectomy, she underwent an uncomplicated Cesarean section. As her calcium levels normalized, plans for femoral fixation were made post-childbirth. One week postpartum, the patient fell, resulting in a pathologic fracture of the left distal femur and ipsilateral iliac wing fracture (Fig. 3A and B).

**Figure 4:**
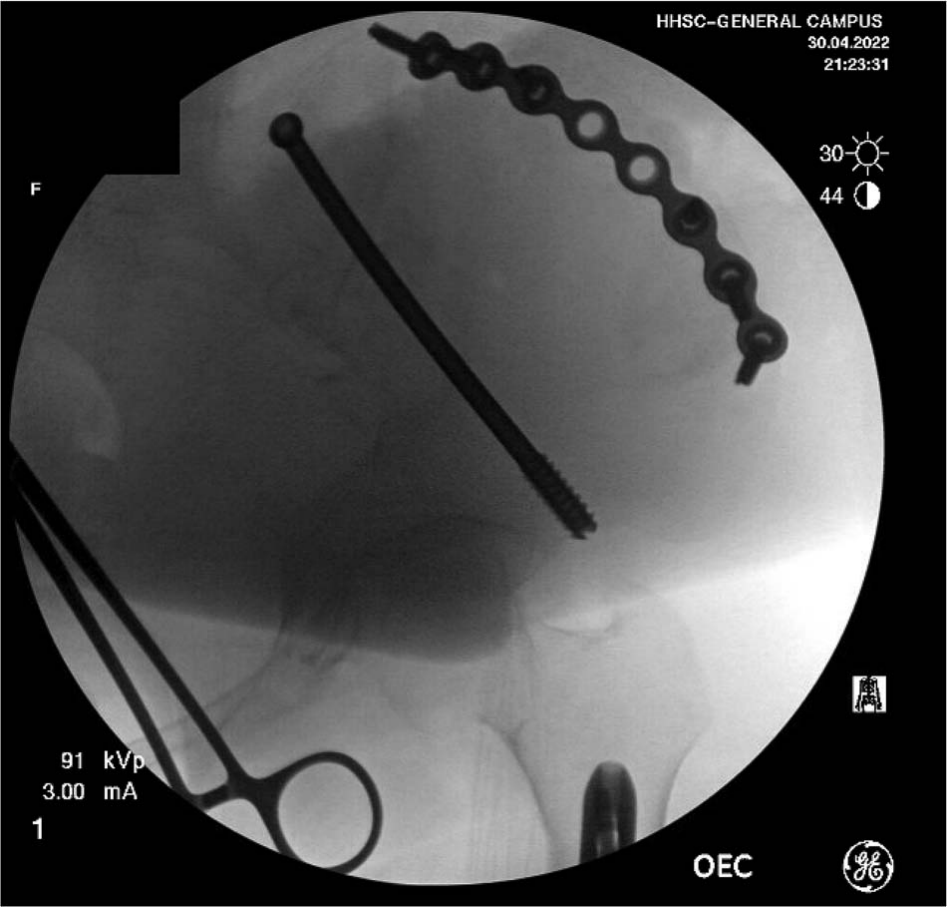
Intraoperative fluoroscopy from ORIF of the pelvis.

Definitive treatment of her distal femur fracture was performed the following day with a reamed, statically locked retrograde intramedullary nail after consultation with the orthopedic oncology team. The procedure was uncomplicated. For the iliac wing fracture, she was transferred to the local level I trauma center 5 days later for surgery by a pelvic and acetabular specialist. The fracture was addressed via a curvilinear approach to the iliac crest, followed by the application of a contoured pelvic reconstruction plate to the outer table of the iliac crest. Fixation was supplemented with an 8.0 mm partially threaded cannulated screw and advanced antegrade through the supraacetabular corridor (Fig. 4).

**Figure 5:**
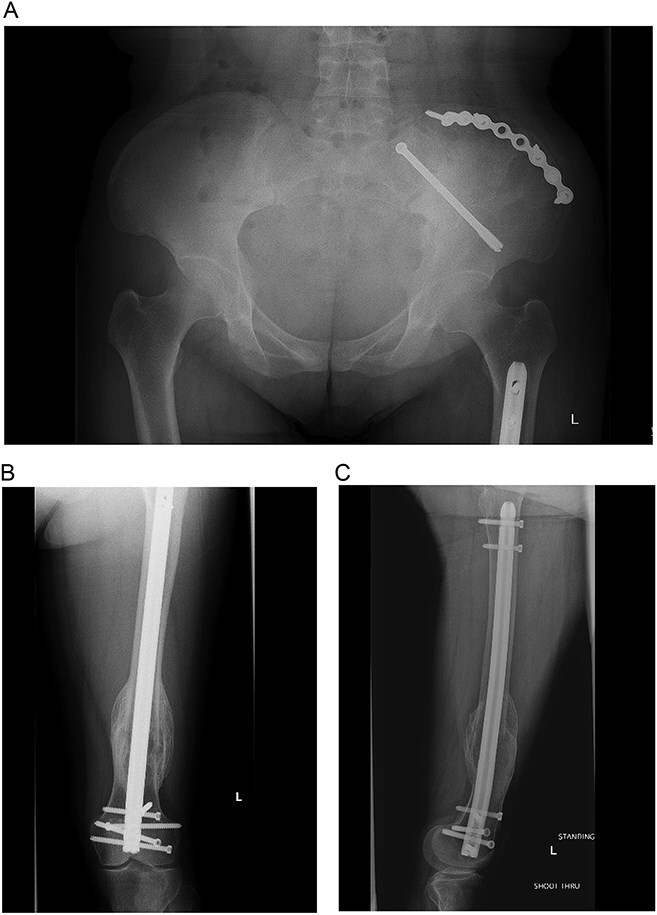
(A–C) Final radiographs showing complete healing >1 year postoperatively.

At the time of pelvic fixation, a bone biopsy confirmed the diagnosis of a brown tumor. The patient underwent routine follow-up and was referred to endocrinology for ongoing calcium and vitamin D deficiency management. At over one year post-op, she had satisfactory clinical and radiographic healing with stable fixation (Fig. 5A–C). A skeletal survey identified additional resolving brown tumors in the right proximal humerus, left distal humerus, and bilateral tibia. By her last follow-up, all lesions had resolved, and she was discharged from orthopedic trauma and oncology care.

## Discussion

This case emphasizes the management of significant pathologic fractures secondary to a brown tumor in the setting of primary hyperparathyroidism, further complicated by pregnancy. Multidisciplinary collaboration was crucial to ensure comprehensive care.

Aggressive bone lesions in young patients raise concern for serious conditions, with the differential diagnosis often including infection, hematologic malignancies, or primary bone sarcomas. Basic laboratory tests should always be part of the initial evaluation. In this case, elevated serum calcium pointed to a brown tumor, highlighting the role of laboratory markers in differentiating benign from malignant conditions. Brown tumors can mimic multiple skeletal metastases, but elevated calcium and PTH levels typically suggest primary hyperparathyroidism as the underlying cause [1, 3]. They may also resemble multiple myeloma in cases of untreated, long-standing hyperparathyroidism [8].

Diagnostic evaluation for bone lesions typically includes biopsy, though diagnostic uncertainty may persist. For instance, Zhong *et al.* [9] described a case where an aggressive bone lesion initially suggested malignancy, with a final diagnosis of brown tumor only after parathyroid hormone testing. Similarly, brown tumors share radiologic and histologic features with GCT [10]. Primary hyperparathyroidism, the third most common endocrine disorder, should always be considered in the differential diagnosis [11, 12]. A thorough history, physical exam, laboratory workup, and biopsy are key for definitive diagnosis.

Although brown tumors are rare, surgical intervention is even less common as these lesions typically regress with the treatment of hyperparathyroidism [13]. In most cases, parathyroidectomy alone resolves the lesions as overactive osteoclastic activity subsides. However, in this case, two significant pathologic fractures required internal fixation to prevent long-term deformity and disability. Reports of internal fixation for pathologic fractures from brown tumors are scarce [14, 15], and none have been documented involving the pelvic ring. This case suggests that surgical intervention is warranted when fractures significantly impair patient independent function or ambulation. Otherwise, lesions can be monitored and typically resolve after treating the underlying hyperparathyroidism.
